# Supplementary material for: Implementation of Antimicrobial Stewardship Programs in Saudi Arabia: A Systematic Review
Source: Microorganisms. 2025 Feb 17;13(2):440. doi: 10.3390/microorganisms13020440 (PMC11858812; doi:10.3390/microorganisms13020440)
Supplement: Supplementary file 1 [file microorganisms-13-00440-s001.zip › microorganisms-3472890-supplementary.pdf]

Table S1: Search Strategy for Identifying Studies on Antimicrobial Stewardship in Saudi Arabia

(TITLE-ABS-KEY ("antimicrob*" OR "antibiot*") AND TITLE-ABS-KEY ("stewardship" OR "stewardship*" OR "asp" OR "ams" OR "resistance" OR "prescribing") AND TITLE-ABS-KEY ("saudi" OR "ksa" OR "riyadh" OR "najran" OR "mecca" OR "makkah" OR "jeddah" OR "qassim" OR "dammam" OR "tabuk" OR "medina" OR "asir" OR "abha" OR "bahah" OR "jawf" OR "jazan" OR "arar" OR "hail" OR "khobar" OR "jubail" OR "yanbu" OR "taif" OR "khafji" OR "al-kharj" OR "al-ahsa" OR "al-majma'ah" OR "al-qunfudhah" OR "al-ula" OR "bisha" OR "diriyah" OR "hafr al-batin" OR "khamis mushait" OR "rabigh" OR "ras tanura" OR "sakaka" OR "sharurah" OR "thuwal" OR "umluj" OR "wadi ad-dawasir")) AND (LIMIT-TO (DOCTYPE , "ar")) AND (LIMIT-TO (LANGUAGE , "english"))

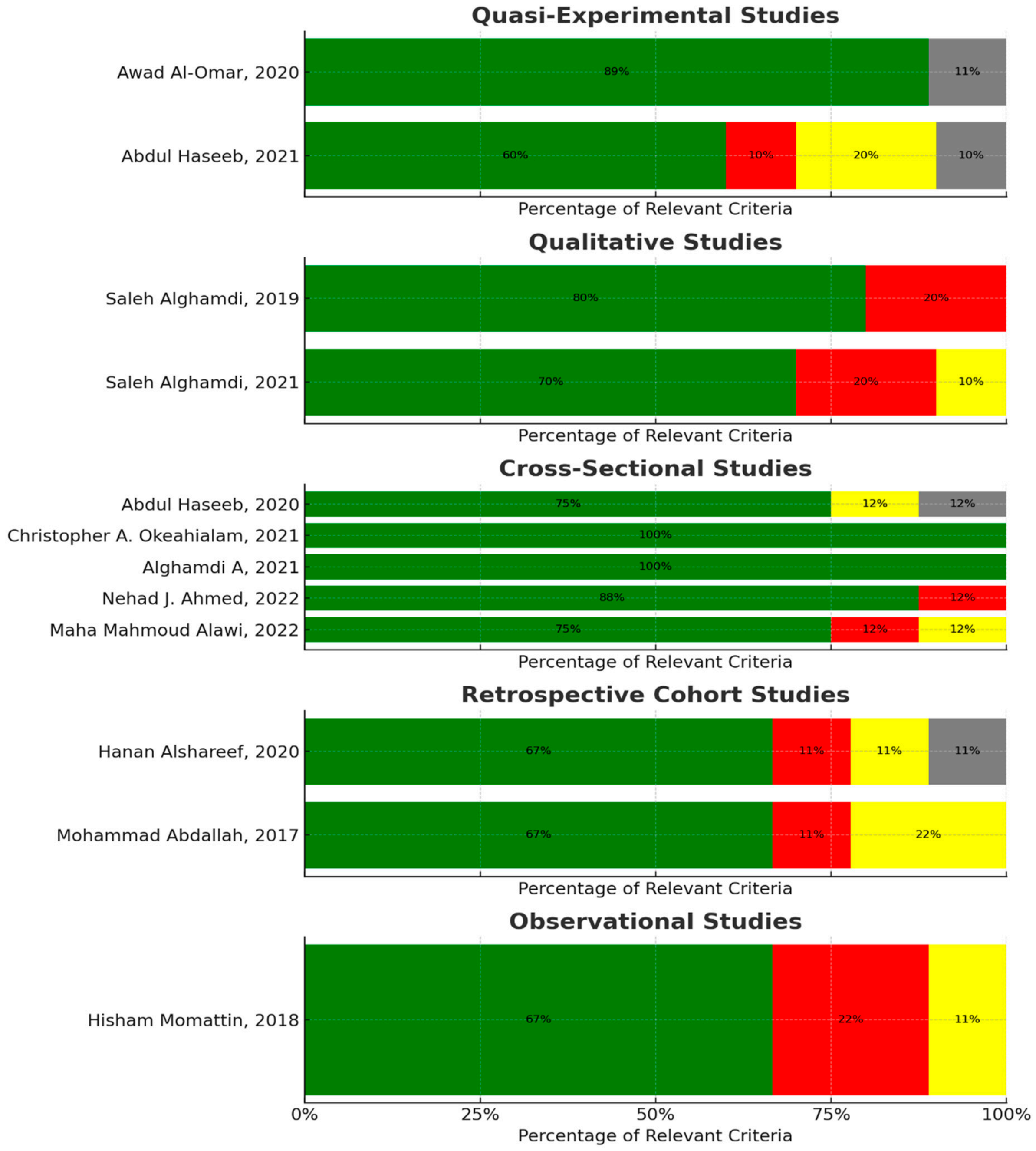

Figure S1. *Summary of methodological rigor assessment across study designs using Joanna Briggs Institute (JBI) criteria.* Bars represent individual studies within each study design category, color-coded by response ("Yes" in green, "No" in red, "Not Clear" in yellow, and "Not Applicable" in gray). Percentage labels within each bar segment provide a clear view of criteria fulfillment, allowing for comparative analysis of methodological rigor across designs.
